# Momordicine I suppresses glioma growth by promoting apoptosis and impairing mitochondrial oxidative phosphorylation

**DOI:** 10.17179/excli2023-6129

**Published:** 2023-06-06

**Authors:** Ying Kao, Chung-Hsing Chou, Li-Chun Huang, Chia-Kuang Tsai

**Affiliations:** 1 Division of Neurosurgery, Department of Surgery, Taipei City Hospital Zhongxing Branch, Taipei 10341, Taiwan; 2 Taipei City University, Taipei 100234, Taiwan; 3 Department of Neurology, Tri-Service General Hospital, National Defense Medical Center, Taipei 11490, Taiwan; 4 Department of Biochemistry, National Defense Medical Center, Taipei 11490, Taiwan

**Keywords:** glioblastoma, Momordica charantia, Momordicine I, oxidative phosphorylation capacity, DLGPA5

## Abstract

Glioblastoma (GBM) is the most common type of primary brain tumor. Patients with GBM have poor survival outcomes. Isolated components of *Momordica charantia* have anticancer effects. However, the bioactivity of *M. charantia* extracts against GBM remains unknown. We tested four major extracts of *M. charantia* and found that momordicine I reduced glioma cell viability without serious cytotoxic effects on astrocytes. Momordicine I suppressed glioma cell colony formation, proliferation, migration, and invasion. Momordicine I also induced apoptosis, intracellular reactive oxygen species (ROS) production, and senescence in glioma cells. Moreover, momordicine I decreased the oxidative phosphorylation capacity of glioma cells and inhibited tumor sphere formation in temozolomide (TMZ)-resistant GBM cells. We further explored whether the antiglioma effect of momordicine I may be related to cell cycle modulation and DLGPA5 expression. Our results indicate that the cytotoxic effect of momordicine I on glioma cells suggests its potential therapeutic application to GBM treatment.

See also Figure 1(Fig. 1).

## Abbreviations

7-AAD 7-aminoactinomycin D

Akt Ak strain transforming (protein kinase B)

BrdU Bromodeoxyuridine

C_12_FDG 5-Dodecanoylaminofluorescein Di-β-D-Galactopyranoside

CGGA Chinese Glioma Genome Atlas

DCF 2',7'-dichlorofluorescein

DCFH-DA 2',7'-dichlorofluorescein diacetate

DLGPA 5 Disks Large Associated Protein 5

DMEM Dulbecco's modified Eagle's medium

DMSO Dimethyl sulfoxide

EDTA Ethylenediaminetetraacetic acid

EMT Epithelial-mesenchymal transition

ER Endoplasmic reticulum

ERK1/2 Extracellular signal-regulated kinase ½

FACS Fluorescence activated cell sorting

FBS Fetal bovine serum

FCCP carbonyl cyanide 4-(trifluoromethoxy)phenylhydrazone

GBM Glioblastoma

GO Gene ontology

MEM Minimum essential medium

MFI Median fluorescence intensity

MGMT O-6-methylguanine-DNA methyltransferase

OCR Oxygen consumption rate

OXPHOS Oxidative phosphorylation

PI Propidium iodide

RIPA buffer Radioimmunoprecipitation assay buffer

ROS Reactive oxygen species

TBST Tris-buffered saline with Tween 20

TMZ Temozolomide

VEGF Vascular endothelial growth factor

## Introduction

Glioblastoma (GBM) is the most common type of malignant primary brain tumor, accounting for almost half of all primary malignant central nervous system tumors (Ostrom et al., 2018[28]). The typical clinical management strategy for patients with GBM is a multimodal approach that includes maximal safe resection, radiotherapy, and temozolomide (TMZ) therapy. However, the median survival time of GBM patients is still less than 15 months even after treatment with surgical resection, concurrent chemoradiotherapy, and adjuvant chemotherapy with TMZ (Ostrom et al., 2018[28]; Wen and Kesari, 2008[44]). Other therapies, such as bevacizumab, a humanized anti-vascular endothelial growth factor (VEGF) antibody, have failed to show a survival benefit in patients with newly diagnosed GBM (Gilbert et al., 2014[13]). Regarding immunotherapies, no innovation with existing immunotherapeutic approaches has been developed for GBM (Lim et al., 2018[24]; Sampson et al., 2020[33]). Therefore, novel therapies for GBM are needed.

*Momordica charantia* (bitter melon, Cucurbitaceae family) is a frequently consumed vegetable in India, Africa, and Southeast Asia (Fang et al., 2019[11]; Krawinkel and Keding, 2006[20]). Some of the isolated components, including momordicine I, kuguacin J, kuguaglycoside C, charantin, α-eleostearic acid, and certain proteins (RNase MC2, MAP30, α-Momorcharin) have effective biological activities (Dandawate et al., 2016[9]). Bitter melon is attracting increasing attention because of its beneficial effects against obesity and type 2 diabetes-associated metabolic disorder (Fan et al., 2019[10]; Leung et al., 2009[21]). The antitumor activity of bitter melon extract was initially validated in mice in 1983 (Jilka et al., 1983[18]). In addition, bitter melon extract or its isolated ingredients have demonstrated noteworthy anticancer activity against skin, prostate, breast, colon, bladder, and pancreatic cancers in several preclinical investigations (Raina et al., 2016[31]).

The extract of *M. charantia* can cross the blood‒brain barrier and exert neuroprotective effects, which has been supported by several animal studies of central nervous system (CNS) diseases, including studies with models of high-fat diet-induced neuroinflammation (Nerurkar et al., 2011[26]), cerebral ischemia (Gong et al., 2015[14]), epilepsy (Li et al., 2018[22]), and Parkinson's disease (Guo et al., 2021[15]).

To determine whether the compounds isolated from *M. charantia *have antiglioma effects, we first chose four major compounds in the *M. charantia* extract, namely, momordicine I, kuguacin J, kuguaglycoside C, and momordicoside I aglycone, for screening experiments. Our data showed that momordicine I inhibited the proliferation, migration, and invasion of GBM cells. In addition, momordicine I induced apoptotic cell death and altered mitochondrial oxidative phosphorylation (OXPHOS). We further investigated whether the antiglioma mechanism of momordicine I is related to cell cycle modification and whether Disks Large-Associated Protein 5 (*DLGAP5*) is the gene encoding the potential downstream target of momordicine I. Considering the results of the current study, we believe that momordicine I treatment has the potential to be a novel therapeutic strategy for glioma.

## Materials and Methods

### Cell culture

LN229 and GBM8401 cells were cultured in Dulbecco's modified Eagle's medium (DMEM) supplemented with 2 % fetal bovine serum (FBS), penicillin, and streptomycin. SVGp12 cells were grown in minimum essential medium (MEM) containing 10 % FBS, penicillin, and streptomycin. The cells were cultured at 37 °C and 5 % CO_2_ to maintain their viability.

### Cell lysate preparation and Western blot analysis

The cells were incubated in RIPA buffer (100 mM Tris-HCl, 150 mM NaCl, 0.1 % SDS, and 1 % Triton X-100) at 4 °C for 10 minutes for lysis. The resulting lysates were then centrifuged at 15,000 rpm for 10 minutes, and the supernatants were collected. Normal brain lysates were procured from Origene Technologies. Subsequently, 30 micrograms of cell lysate from each group were loaded onto a 10 % sodium dodecyl sulfate-polyacrylamide gel for electrophoresis. The proteins were transferred onto polyvinylidene fluoride membranes (Millipore, MA, USA), and then the membranes were blocked with 5 % skim milk in TBST at room temperature for 1 hour. Antibodies were purchased from Cell Signaling Technology (Danvers, MA, USA; anti-N-cadherin), Abcam (Cambridge, MA, USA; anti-survivin, anti-O-6-methylguanine-DNA methyltransferase (MGMT)), Agilent (Santa Clara, CA, USA; anti-Ki-67), and Atlas Antibodies AB (Bromma, Sweden; anti-DLGAP5). The antibodies were diluted to the specified ratios using enhancer kits in accordance with the manufacturer's guidelines (anti-survivin: 1:1000; anti-Ki-67: 1:250; anti-N-cadherin: 1:1000; anti-DLGAP5: 1:1000; anti-MGMT: 1:2000). The bands were visualized by employing enhanced chemiluminescence reagents and X-ray film (GE Healthcare, Piscataway, NJ, USA).

### Cell viability assay

To gauge cell viability, 3×10^3^ LN229, GBM8401, and SVGp12 cells/well were plated in 96-well plates. The next day, the cells were treated with momordicine I, kuguacin J, kuguaglycoside C, and momordicoside I aglycone at specific concentrations for 48 h. To conduct the CellTiter 96 Aqueous One Solution Cell Proliferation Assay (G3581, Promega, Madison, WI, USA), twenty microliters of MTS reagent were added to the cells, and they were incubated for 2-4 hours based on the manufacturer's instructions. Next, the absorbance at 490 nm was quantified with a Varioskan LUX multimode microplate reader (Thermo Scientific, Waltham, MA, USA) (Tsai et al., 2019[41]).

### Colony formation assay

LN229 and GBM8401 cells (1×10^3^) were plated in 6-well plates. The next day, the cells were treated with momordicine and incubated at 37 °C and 5 % CO_2_ for 14 days before being stained with crystal violet. To quantify clonogenic cell growth, ImageJ software (NIH, Bethesda, MD) was used, and colonies larger than 0.5 mm in diameter were counted.

### Cell proliferation, cell cycle, cellular apoptosis, cellular senescence, intracellular reactive oxygen species (ROS) generation, and flow cytometry assays

To evaluate cell proliferation, LN229 and GBM8401 cells (5×10^4^ cells in 6-well plates) were treated with momordicine I for 48 hours at the specified concentration and processed with FITC-BrdU Flow Kits following the instructions provided by the manufacturer (BD Biosciences). For the assessment of cell cycle distribution after momordicine I treatment, the DNA content was measured via fluorescence-activated cell sorting (FACS) as described previously (Pozarowski and Darzynkiewicz, 2004[30]). The experimental and control groups were both treated with 70 % ethanol at 4 °C and subsequently stored at -20 °C overnight. Subsequently, the cells were rinsed twice with chilled phosphate-buffered saline (PBS) and stained in the dark with a propidium iodide (PI) solution (consisting of 50 μg/ml PI in PBS, 1 % Tween 20, and 10 μg/ml RNase A) for 30 minutes. The DNA content was then quantified by FACS (BD Biosciences, San Jose, CA, USA) in two separate experiments.

To analyze cellular apoptosis, a 7-AAD/ PE Annexin V assay was conducted in accordance with a previous description (Andree et al., 1990[1]; Casciola-Rosen et al., 1996[4]; Chang et al., 2017[6]; Vermes et al., 1995[42]). In brief, cells were seeded in 6-well plates. The next day, the cells were treated with the indicated concentration of momordicine I for 48 h. Then, the cells were collected, and the protocol was performed in accordance with the instructions from the manufacturer (BD MitoScreen). A FACSCalibur flow cytometer (BD Biosciences) and Cell-Quest Pro software (BD Biosciences) were utilized to examine all samples.

The intracellular production of ROS was assessed using a 2',7'-dichlorofluorescein diacetate (DCFH-DA) assay. We incubated the momordicine I-treated cells with 10 mM DCFH-DA at 37 °C for 30 min. Then, we harvested the cells and washed them twice with PBS. The 2',7'-dichlorofluorescein (DCF) fluorescence was analyzed by using a FACSCalibur flow cytometer (BD Biosciences) (Cheng et al., 2016[8]). To analyze cellular senescence via flow cytometry, 5-dodecanoylaminofluorescein di-β-D-galactopyranoside (C_12_FDG; D2893, Invitrogen, Thermo Fisher Scientific, Waltham, MA, USA) was utilized as a substrate, which emits fluorescence and becomes membrane impermeable when cleaved by β-galactosidase. LN229 and GBM8401 cells were dissociated with trypsin-EDTA, washed with PBS, and incubated with 33 μM C_12_FDG in 50 μl of PBS for 1 hour. Then, the cells were analyzed immediately using a FACSCalibur flow cytometer, and the fluorescence signal of C_12_FDG was measured in an FL1 detector. The β-galactosidase activity was presented as the median fluorescence intensity (MFI) of the glioma population, and data were evaluated using FACSDiva software (Version 6.1, BD Biosciences, San Jose, CA, USA) according to previously described methods (Noppe et al., 2009[27]; Tsai et al., 2018[40]).

### Global gene expression profiling and biological pathway analysis

We analyzed total RNA from LN229 and GBM8401 cells with Human OneArray Plus microarrays (Phalanx Biotech Group, Hsinchu, Taiwan). Then, we determined gene expression using Agilent Technologies (Santa Clara, CA, USA) 0.1 XDR protocol. Fold changes were calculated as the ratios of the mean values in momordicine I-treated cells to the values in untreated cells. Then, the significant differentially expressed genes were subjected to Gene Ontology (GO) Biological Process analysis, which is an effective method for annotating genes and gene products and for revealing characteristic biological attributes based on high-throughput genomic or transcriptomic data (Gene Ontology Consortium, 2006[12]; Ashburner et al., 2000[2]). The mRNA microarray data are available at the NCBI Gene Expression Omnibus (accession no. GSE231550).

### Bioinformatics analysis using the Chinese Glioma Genome Atlas (CGGA) dataset

We used the CGGA (http://wwwcgga.org.cn) (Zhao et al., 2021[48]) online tool to perform customizable functional analyses, such as patient survival analysis, cancer and normal tissue differential expression analyses, and gene correlation analyses.

### Measurement of the oxygen consumption rate (OCR)

To measure cellular oxidative respiration, a Seahorse XF bioenergetic system was utilized in combination with a Seahorse Cell Mito Stress Test Kit (Seahorse Bioscience, North Billerica, MA, USA). LN229 and GBM8401 glioma cells were seeded into an XFp microplate in DMEM supplemented with 2 % FBS and treated with momordicine for 24 hours over the following two days. Next, the medium was replaced with sodium bicarbonate-free DMEM supplemented with 2 % FBS, and the OCR was measured at steady state. To determine the maximal and nonmitochondrial respiration rates, oligomycin (1 μM), carbonyl cyanide 4-(trifluoromethoxy)phenylhydrazone (FCCP; 0.5 μM), and a mixture of rotenone (0.5 μM) and myxothiazol (1 μM) were automatically added to the wells, as per the manufacturer's instructions, as part of the Seahorse Cell Mito Stress Test. 

### Statistical analysis

Differences between the experimental and control groups were estimated by Student's t test. Outcomes are reported as means ± SDs or as stated. Statistical significance was set at P less than 0.05.

## Results

### Momordicine I decreases the viability of glioma cells without serious cytotoxic effects on astrocytes

**Figure 2:**
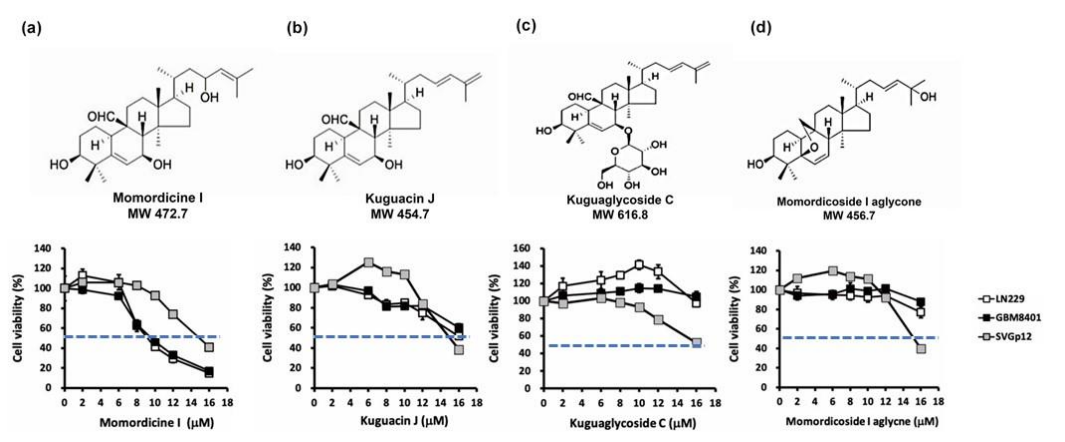
Cytotoxic effect of isolated *M. charantia* L. compounds on SVGp12 astrocytes and LN229 and GBM8401 glioma cells. (a-d, top panels) Molecular structure and molecular weight of each tested compound. SVGp12 astrocytes, LN229 glioma cells, and GBM8401 glioma cells were treated with momordicine I, kuguacin J, kuguaglycoside C, and momordicoside I aglycone for 48 h. Among the compounds, momordicine I exerted the strongest cytotoxic effect on LN229 and GBM8401 glioma cells in the MTS assay. All experiments were performed three times. The blue line indicates the IC_50_. The data are presented as means ± standard errors of the means (SEMs).

To validate the antiglioma effect of isolated compounds in *M. charantia* L. extract, we purchased momordicine I, kuguacin J, kuguaglycoside C, and momordicoside I aglycone from ChemFaces Biotechnology (Wuhan, Hubei 430056, PRC). The molecular structure of each tested compound is shown in Figure 2a-d(Fig. 2) (top panels). To assess the cytotoxic properties of these four compounds in LN229 and GBM8401 glioma cells, we used an MTS assay to evaluate cell viability after treatment with specific concentrations for 48 h. SVGp12 astrocytes were used as normal controls. As shown in Figure 2a-d(Fig. 2) (bottom panels), momordicine I dose-dependently suppressed the proliferation of GBM8401 and LN229 glioma cells (Figure 2a(Fig. 2)). However, kuguacin J, kuguaglycoside C, and momordicoside I aglycone did not suppress the proliferation of GBM8401 and LN229 glioma cells (Figure 2b-d(Fig. 2)). The half-maximal inhibitory concentration (IC_50_) of each compound in SVG-p12 astrocytes, GBM8401 glioma cells, and LN229 glioma cells is listed in Table 1(Tab. 1). Although high concentrations of momordicine I reduced astrocyte viability, no significant cytotoxic effects were observed on SVG-p12 astrocytes. Based on these results, we concluded that momordicine I exerted an inhibitory effect on glioma cell proliferation without serious cytotoxic effects on astrocytes.

### Momordicine I suppresses colony formation by human glioma cells

**Figure 3:**
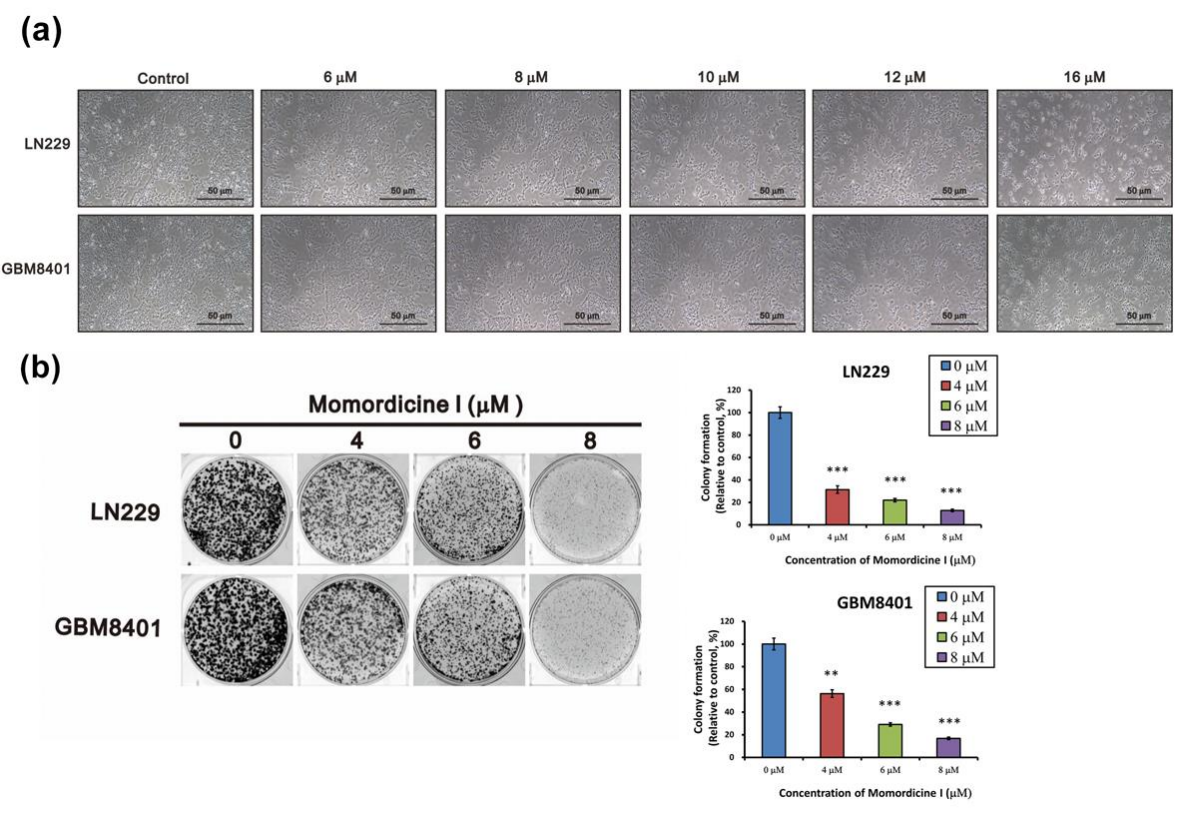
Momordicine I suppresses colony formation by LN229 and GBM8401 cells. (a) Representative images of LN229 and GMB8401 glioma cells in the absence or presence of the indicated concentrations of momordicine I. (b) Glioma cells were incubated with DMSO or different concentrations of momordicine I for 14 days. The results of quantitative analysis of colony formation are shown as mean ± SEM of 3 independent experiments. For comparison between two means, Student's unpaired *t*-test was used. ** *p*<0.01; *** *p*<0.001.

To understand the morphological change in glioma cells in the presence of momordicine I, we observed cell morphology after treatment with specific concentrations of momordicine I 48 h after treatment. The percentages of viable LN229 and GBM8401 cells decreased as the concentration of momordicine I increased (Figure 3a(Fig. 3)). To assess the long-term antiglioma effect of momordicine I, we performed colony formation assays using LN229 and GBM8401 cells treated with momordicine I. The findings revealed that momordicine I inhibited colony formation in both GBM cell lines in a concentration-dependent manner (Figure 3b(Fig. 3)).

### Momordicine I suppresses proliferation and induces apoptotic death in human glioma cells

**Figure 4:**
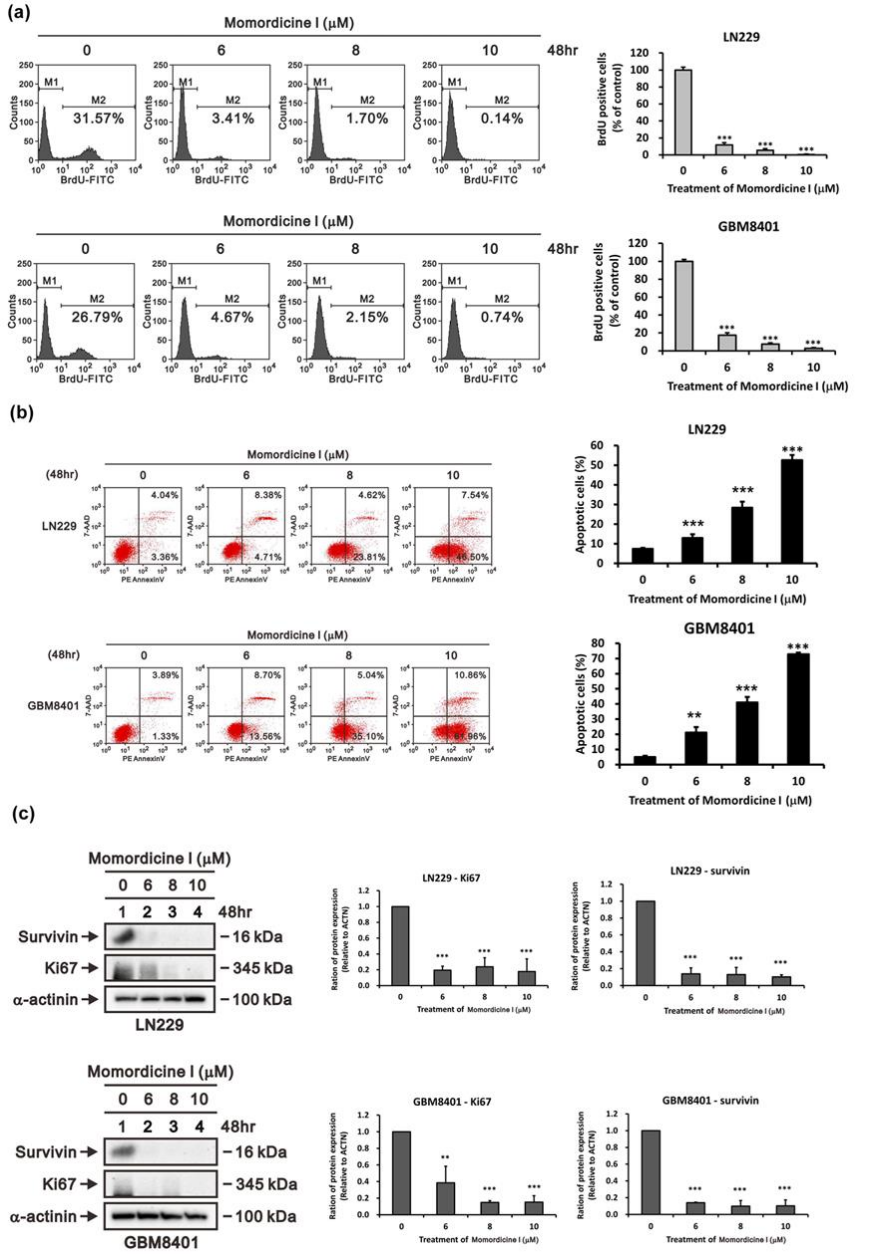
Momordicine I reduced proliferation and induced apoptosis in GBM cell lines. (a) After 48 hours of incubation with momordicine I, glioma cells were pulsed with BrdU and subsequently subjected to flow cytometric analysis. M1, BrdU-negative cells; M2, BrdU-positive cells. Cells not pulsed with BrdU were used as a blank control. The results are presented as means ± SEMss (n=3); *** p<0.001. (b) Apoptosis assay of glioma cells treated with momordicine I by 7-AAD/PE Annexin V staining. The apoptotic cells (early and late stages) were identified by flow cytometry, and apoptosis rates are presented as percentages. The results are presented as means ± SEMss (n=3); ** p<0.01; *** p<0.001. (c) LN229 and GBM8401 glioma cells were treated with the indicated concentrations of momordicine I for 48 h; whole-cell lysates were prepared and subjected to Western blot analysis of survivin and Ki-67 expression. α-Actinin served as loading control. The results are presented as means ± SEMss (n = 3); ** p<0.01; *** p<0.001.

To further examine the impact of momordicine I on cell proliferation, we performed a BrdU incorporation assay with flow cytometry. As shown in Figure 4a(Fig. 4), momordicine I dose-dependently inhibited proliferation in both glioma cell lines, as verified by BrdU flow cytometry analysis (with a decrease in the number of proliferating LN229 cells from 31.57 % to 0.14 % and 26.79 % to 0.74 % in GBM8401 cells). In addition, crude extracts of bitter melon induced apoptotic death in various cancer cell lines. Thus, we tested whether momordicine I induces apoptotic death in LN229 and GBM8401 cells by 7-AAD/PE Annexin V staining with flow cytometry (Figure 4b(Fig. 4)). Treatment with momordicine I (6-10 μM) for 48 h dose-dependently induced apoptotic death in both LN229 and GBM8401 cells (with an increase in the apoptosis rate from 7.40 % to 54.04 % in LN229 cells and 5.22 % to 72.82 % in GBM8401 cells). Moreover, we evaluated the expression of survivin and Ki-67 in LN229 and GBM8401 cells after momordicine I treatment by immunoblotting. Momordicine I suppressed the expression of these proteins (Figure 4c(Fig. 4)). These results indicated that momordicine I inhibited proliferation and induced apoptotic death in glioma cell lines.

### Momordicine I impedes the migration and invasion of LN229 and GBM8401 cells via alterations in the expression of epithelial-mesenchymal transition (EMT) markers

**Figure 5:**
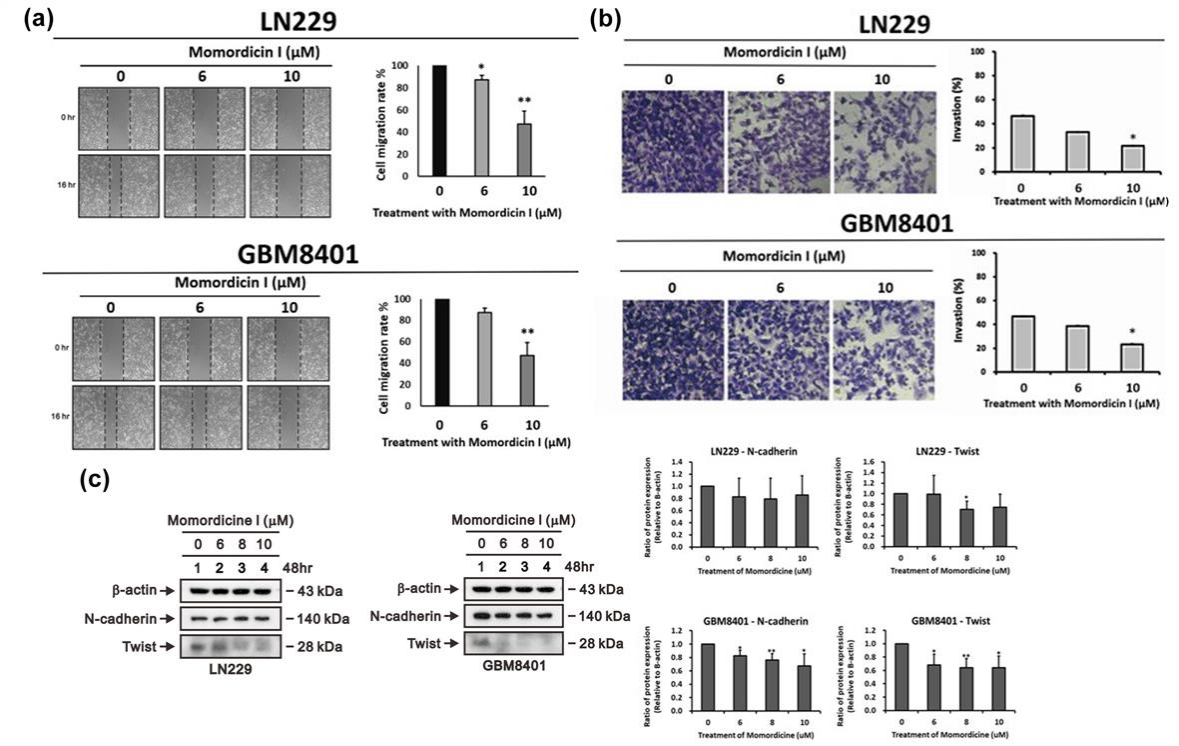
Momordicine I suppresses the migration of LN229 and GBM8401 GBM cells. (a) Scratches were made in LN229 and GBM8401 glioma cell monolayers in 6-well plates, and the cells were cultured in the presence or absence of momordicine I for 16 h. Momordicine I-treated (6 and 10 µM) cells had a significantly lower wound area relative than the control group in both LN229 and GBM8401 cells. All cells were pretreated with mitomycin C, a cell cycle inhibitor, to assess migration without the confounding impact of cell proliferation. The wound area was analyzed with ImageJ software, and the data are expressed relative to hour 0 (n≥3; * p<0.05; ** p<0.01.) (b) The invasive activity of LN229 and GBM8401 cells was measured by Transwell invasion assays. The results are presented as means ± SEMss (n≥3; * p<0.05). (c) LN229 and GBM8401 glioma cells were treated with the indicated concentrations of momordicine I for 48 h; whole-cell lysates were prepared and subjected to Western blotting and quantitative analysis, and the expression of N-cadherin, and Twist was normalized to that of the internal control (n=3; * p<0.05; ** p<0.01).

Aggressive invasion and migration are important features of GBM and cause poor differentiation between normal brain tissue and tumor tissue. Therefore, we tested whether momordicine I has inhibitory effects on the migration and invasion of glioma cells. The results of the wound healing assay and transwell invasion assay confirmed that momordicine I suppressed human LN229 and GBM8401 glioma cell migration (Figure 5a(Fig. 5)) and invasive ability (Figure 5b(Fig. 5)). Moreover, a reduction in the expression of the mesenchymal marker Twist was found in momordicine I-treated glioma cells (Figure 5c(Fig. 5)).

### Momordicine I increases intracellular ROS generation and senescence in glioma cells

**Figure 6:**
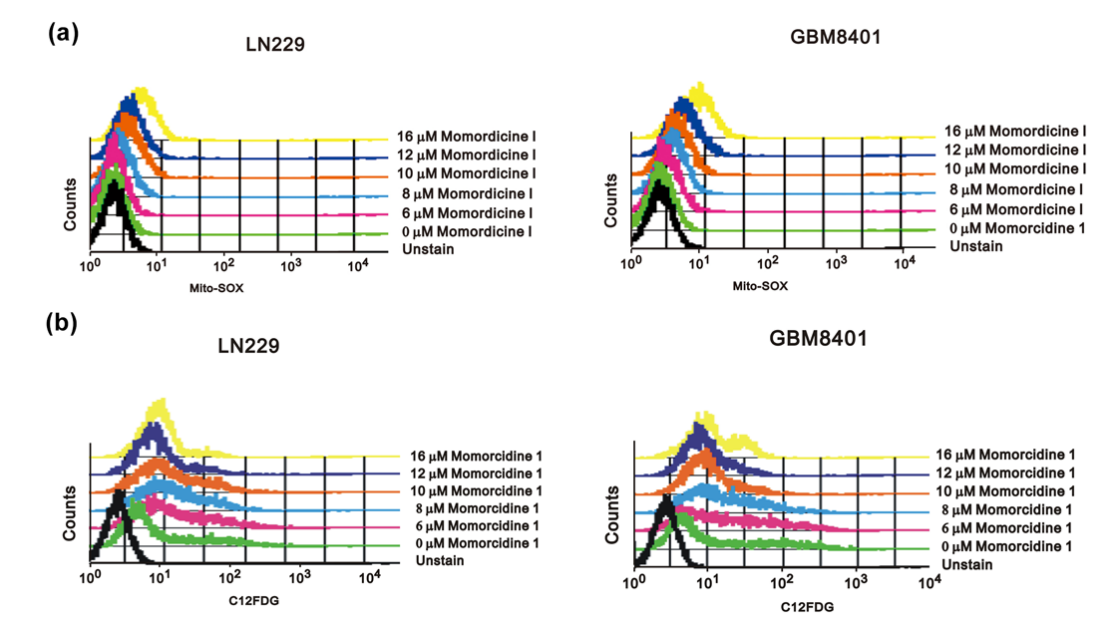
Momordicine I increased intracellular ROS generation and senescence in glioma cells. (a) LN229 and GBM8401 cells were cultured with specific concentrations of momordicine I for 30 min. Cellular ROS levels were measured using Mito-SOX staining and flow cytometry. Unstained cells served as blank controls. (b) Cells were incubated for 30 min with the indicated concentrations of momordicine I and were then stained with C_12_FDG and assayed by flow cytometry. Cells without C_12_FDG treatment served as blank controls.

*M. charantia* extracts can induce ROS-mediated cell death in human lung cancer and ovarian cancer (Chan et al., 2020[5]; Thiagarajan et al., 2019[39]). To understand the effect of momordicine I on ROS generation in glioma, we used Mito-SOX staining with flow cytometry. As shown in Figure 6a(Fig. 6), momordicine I dose-dependently increased intracellular ROS production. Furthermore, we performed a C_12_FDG assay to determine whether momordicine I induces senescence in glioma cells. Senescence was enhanced in LN229 and GBM8401 cells treated with momordicine I (Figure 6b(Fig. 6)). Based on these results, we concluded that momordicine I increased intracellular ROS generation and senescence in glioma cells.

### Momordicine I reduces the OXPHOS capacity of glioma cells

**Figure 7:**
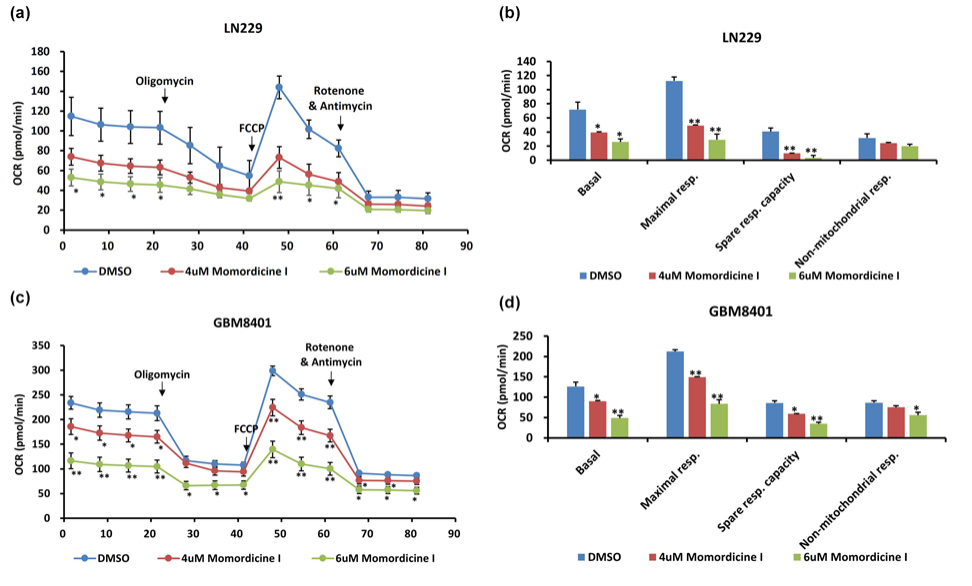
Momordicine I regulates mitochondrial OXPHOS. The Seahorse XFe48 Extracellular Flux Analyzer was utilized to conduct bioenergetic analysis on glioma cells treated with momordicine I and DMSO as a control. CR values are plotted (a, c). Data are plotted to demonstrate the differences in the basal respiration rate, maximal respiratory capacity, spare respiratory capacity, and non-mitochondrial respiration rate (b, d). * p<0.05; ** p<0.01; *** p<0.001; n=2.

To understand the relationship between momordicine I and the metabolic profile of glioma cells, we used the Seahorse bioanalyzer to test whether momordicine I affects the OXPHOS capacity of glioma cells. Significant decreases in OXPHOS-related parameters, including the OCR, basal respiration rate, maximal respiratory capacity, and nonmitochondrial respiration rate, were observed in both LN229 (Figure 7a, b(Fig. 7)) and GBM8401 (Figure 7c, d(Fig. 7)) cells treated with momordicine I. These data show that momordicine I regulates the OCR and mitochondrial respiration. Thus, its antiglioma effect could be partially mediated through metabolic dysregulation.

### Momordicine I overcomes the tumorigenicity of TMZ-resistant (TMZ-R) GBM cells

**Figure 8:**
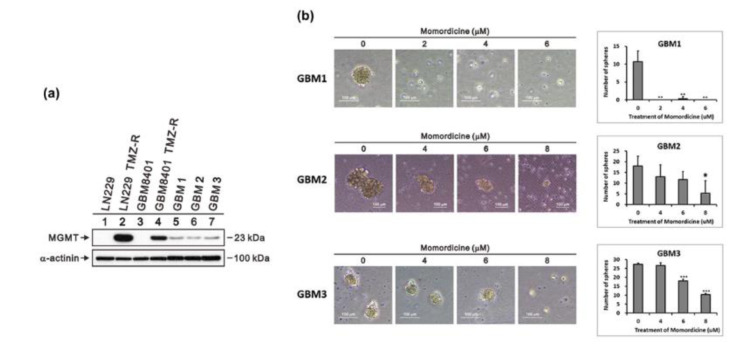
Momordicine I suppressed tumor sphere formation. Representative images of spheres formed by temozolomide-resistant GBM cells derived from clinical specimens. (a) MGMT protein levels in GBM8401 cells, LN229 cells, and cells from human clinical GBM samples. Whole-cell lysates were prepared and subjected to Western blotting. α-Actinin served as loading control. (b) Cells were incubated with the indicated concentrations of momordicine I in ultralow-attachment 6-well plates for 14 days. The data are presented as means ± SDss (n=3). Scale bars, 100 μm. * p<0.05.

GBM cells acquire chemoresistance to TMZ by expressing a DNA repair enzyme, MGMT. This is an important factor linked to the poor survival of patients with glioma (Hegi et al., 2005[16]). The development of drug resistance is associated with metabolic reprogramming (Strickland and Stoll, 2017[35]). Since momordicine I decreased the OXPHOS capacity of glioma cells, we further tested whether momordicine I can inhibit the growth of TMZ-resistant GBM cells derived from clinical samples (GBM1, GBM2, GBM3). As shown in Figure 8a(Fig. 8), we measured the expression of the DNA repair enzyme MGMT to assess whether the glioma cells had TMZ resistance. MGMT expression was higher in TMZ-resistant LN229 and GBM8401 cells and in GBM1, GBM2, and GBM3 cells than in control LN229 and GBM8401 cells. Next, we used a tumor sphere formation assay to test whether momordicine I inhibits the growth of TMZ-resistant GBM cells. As shown in Figure 8b(Fig. 8), momordicine I significantly suppressed tumor sphere formation by TMZ-resistant human GBM cells. Based on these data, momordicine I could overcome the tumorigenicity of TMZ-resistant GBM cells.

### The antiglioma effects of momordicine I on glioma cells may be related to cell cycle modification and DLGPA5 expression

**Figure 9:**
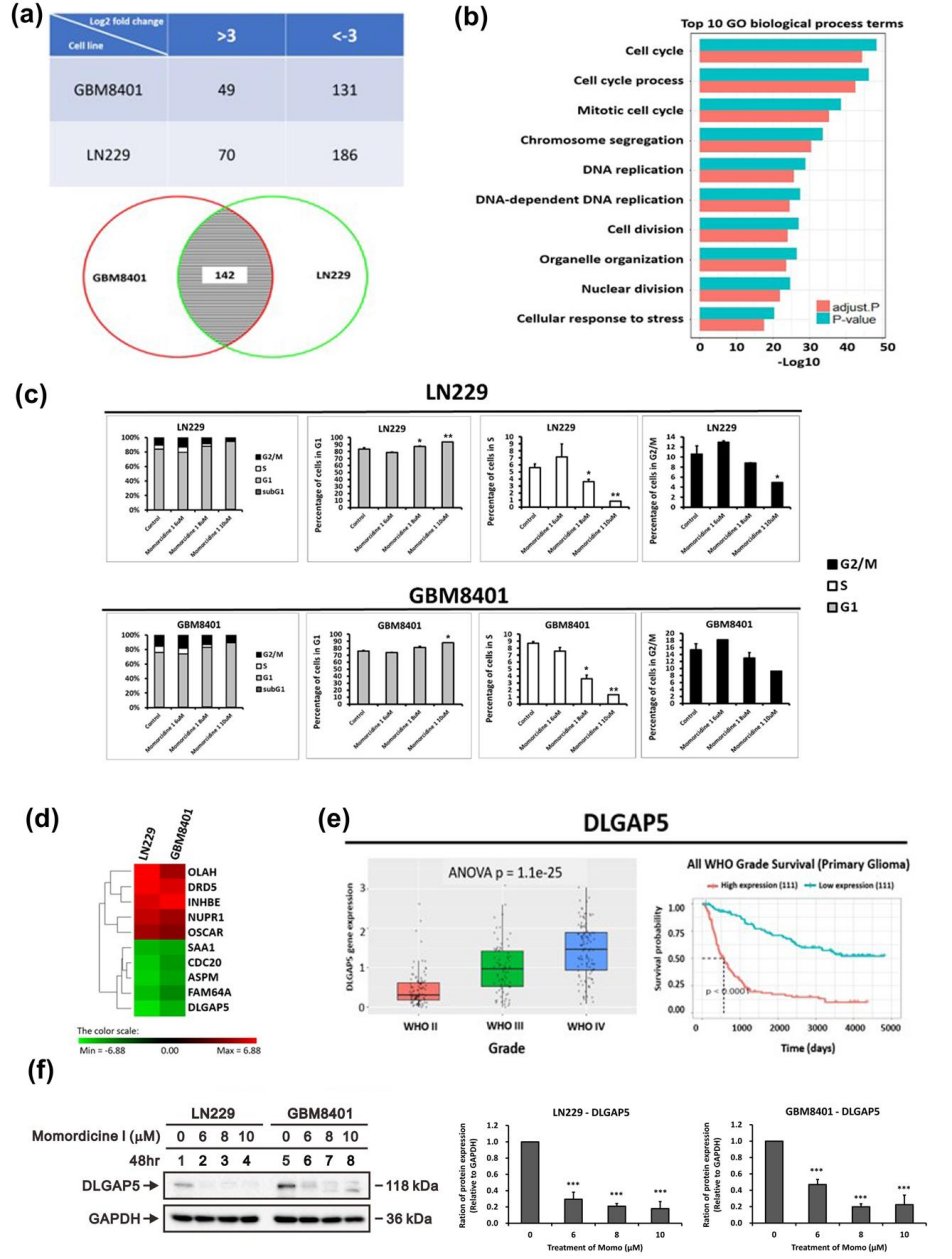
Genes exhibiting differential expression in LN229 and GBM8401 cells following a 48-hour incubation with momordicine I. (a) Numbers of differentially expressed genes with a greater than 3-fold change in expression in LN229 cells and GBM8401 (top panel). Venn diagram showing that the expression of 142 genes was affected by momordicine I in both glioma cell lines (bottom panel). (b) List of the top 10 enriched biological process terms associated with momordicine I treatment. The gene expression data were analyzed with GO enrichment analysis tools. (c) Cell cycle analysis was conducted on LN229 and GBM8401 cells treated with momordicine I by PI staining and flow cytometry. The data are expressed as means ± SDss; n=2; * p<0.05, ** p<0.01. (d) Heatmap showing the top 5 upregulated and downregulated mRNAs. (e) Quantification of DLGAP5 mRNA expression in patients with WHO grade II to grade IV tumors (e, left panel) and Kaplan-Meier survival analysis (e, right panel) based on DLGAP mRNA expression in the CGGA RNA sequencing dataset. (f) DLGAP5 protein levels in GBM8401 and LN229 cells treated with 6, 8, and 10 μM momordicine I for 48 h. Whole-cell lysates were prepared and subjected to Western blotting. The results are presented as means ± SEMss (n=3); *** p<0.001.

The preliminary results verified that momordicine I has antiglioma effects. To elucidate the downstream molecular pathways, we utilized gene microarray analysis to identify the downstream targets and pathways most strongly correlated with this effect. LN229 and GBM8401 cells cultured with momordicine I for 48 h were investigated. Differentially expressed genes were identified as those with a greater than 3-fold difference in expression between momordicine I-treated and untreated cells; 70 upregulated and 186 downregulated genes were identified in LN229 cells, and 49 upregulated and 131 downregulated genes were identified in GBM8401 cells (Figure 9a(Fig. 9), top panel). The Venn diagram demonstrates that the expression of 142 genes was influenced by momordicine I in both glioma cell lines (Figure 9a(Fig. 9), bottom panel). Next, we used GO enrichment analysis to identify the top 10 enriched biological process terms, which included cell cycle, cell cycle process, and mitotic cell cycle (Figure 9b(Fig. 9)). Cell cycle analysis by flow cytometry revealed that momordicine I led to G1 arrest in GBM cells (Figure 9c(Fig. 9)). The top 5 up- and downregulated genes are shown in Figure 6d(Fig. 6). After reviewing the literature and searching the CGGA (http://cgga.org.cn/, accessed on 25 December 2021), we confirmed that DLGAP5 expression was correlated with advanced grade and poor survival in glioma patients (Figure 9e(Fig. 9)). Momordicine I suppressed DLGAP5 protein expression in LN229 and GBM8401 glioma cells (Figure 9f(Fig. 9)), implying that this protein is the likely downstream target. These results indicated that momordicine I inhibited glioma cell survival through cell cycle modulation and that DLGAP5 is probably the downstream target of momordicine I in glioma cells.

See also the Supplementary data.

## Discussion

In this study, we found that momordicine I could decrease the viability and inhibit the proliferation and migration of glioma cells through cell cycle modulation. Momordicine I increased intracellular ROS accumulation and promoted apoptosis. Moreover, momordicine I regulated the OCR and mitochondrial respiration. We also found that momordicine I could overcome the tumorigenicity of TMZ-resistant GBM cells. Finally, we revealed that the antiglioma effect of momordicine I may be related to decreased DLGAP5 expression and cell cycle disturbance.

Extracts from *M. charantia* have anticancer activity, and the effects of several isolated components on glioma cells have been examined. First, Wang et al. reported that charantagenin D, a compound derived from the fruit of *M. charantia* L., showed significant cytotoxic effects on U87 cells (Wang et al., 2012[43]). Second, Manoharan et al. indicated that the protein α, β momorcharin, isolated from *M. charantia*, reduced the viability of 1321N1 astrocytoma and U87-MG glioma cells by increasing apoptotic activity through a mechanism involving augmented caspase-3 and 9 expression and cytochrome c release (Manoharan et al., 2014[25]). Third, Jiang et al. reported that Momordica anti-human immunodeficiency virus protein of 30 kDa (MAP30), a protein isolated from *M. charantia*, inhibited the growth and invasion of U87 and U251 cells in a dose- and time-dependent manner. They also discovered that MAP30 induced apoptosis by inhibiting the Wnt/β-catenin signaling pathway (Jiang et al., 2018[17]). Our study is the first to determine that one of the compounds isolated from *M. charantia* extracts, momordicine I, exerts an antiglioma effect related to cell cycle modulation, inhibited DLGAP5 expression, and suppressed OXPHOS activity. Further *in vivo* experiments should be conducted to clarify the therapeutic effect of momordicine I in glioma treatment.

Metabolic reprogramming contributes to the development of drug resistance (Strickland and Stoll, 2017[35]). The predominant metabolic shift that enables cancer cells to exhibit prolonged survival is the adjustment to aerobic glycolysis, which is characterized by low oxygen consumption, high lactate production, and high glucose uptake (Poteet et al., 2013[29]; Strickland and Stoll, 2017[35]). Thus, discovering metabolic weaknesses in cancer cells and strategies to target these weaknesses is an approach to overcome therapeutic resistance in GBM. Sur et al*.* reported that bitter melon extracts inhibited glycolysis and lipid metabolism in oral cancer. Additionally, they induce ER stress and oxidative stress-mediated cell death (Sur et al., 2019[36]). In this study, we discovered that momordicine I increased intracellular ROS production and significantly decreased the OCR in glioma cells. Moreover, Somasagara et al. found that bitter melon juice decreased the viability of gemcitabine-resistant pancreatic cancer cells by inhibiting the phosphorylation of Akt and ERK1/2, which are crucial in the chemoresistance of pancreatic cancer (Somasagara et al., 2015[34]). Our data revealed that momordicine I inhibited the tumorigenicity of TMZ-resistant GBM cells. This is the first study to reveal that momordicine I has the potential to modify glioma cell metabolism and to overcome the tumorigenicity of chemoresistant GBM cells. More research is needed to determine the targets of momordicine I in glioma cell metabolic reprogramming and drug resistance.

The DLGAP5 protein, also called HURP or DLG7, is crucially involved in cell cycle regulation. DLGAP5 mRNA was found to be present during S phase and to persist during G2 and M phases (Bassal et al., 2001[3]). During mitosis, DLGAP5 mediates spindle stability and dynamics to enable efficient kinetochore capture and proper interkinetochore tension, further promoting chromosomal congression (Wong and Fang, 2006[45]; Wong et al., 2008[46]). Many studies have identified the relationship between DLGAP5 and cancers. The expression of DLGAP5 has been found to be upregulated in several cancers, such as endometrial cancer (Chen et al., 2023[7]), ovarian cancer (Zhang et al., 2021[47]), bladder cancer (Rao et al., 2022[32]), hepatocellular cancer (Liao et al., 2013[23]), pancreatic cancer (Ke et al., 2020[19]), and lung cancer (Tagal et al., 2017[37]). Pancancer analysis revealed that *DLGAP5* was highly expressed in most cancers and that high *DLGAP5* expression was significantly correlated with poor prognosis in cancer patients (Tang et al., 2021[38]). The expression of DLGAP5 was also found to be elevated in glioma. Glioma patients with high *DLGAP5* expression had poor survival outcomes, and silencing *DLGAP5* inhibited glioma cell proliferation (Zhou et al., 2021[49]). Our data showed that momordicine I can induce cell cycle arrest and inhibit DLGAP5 expression in glioma cells, indicating that momordicine I has therapeutic potential in glioma treatment. However, the detailed mechanism by which momordicine I regulates DLGAP5 expression requires further study.

## Conclusion

In summary, momordicine I reduced the viability and inhibited the proliferation and migration of glioma cells. Momordicine I induced cell cycle arrest by targeting DLGAP5 in glioma cells. Furthermore, momordicine I exhibited the potential to reprogram glioma cell metabolism and to suppress the tumorigenicity of TMZ-resistant GBM cells. Considering the poor survival outcomes of GBM patients, our novel results have translational potential in treating this malignant CNS tumor.

## Declaration

### Author contributions

Kao and Tsai conceived and designed this research. Kao, Chou, Huang and Tsai collected the data. Kao, Huang, and Tsai carried out the experiments. Kao, Chou, Huang, and Tsai analyzed the results. Kao and Tsai wrote the first draft of the paper. Tsai revised the paper. All authors read and approved the final manuscript.

### Data availability statement

The data that support the findings of this study are available on request from the corresponding author.

### Funding

The study was funded by the Ministry of Science and Technology of Taiwan (grant numbers MOST 110-2314-B-016-035 and 111 2314-B-016-054), the Ministry of National Defense Medical Affairs Bureau (MND-MAB-D-112107), and the Tri-Service General Hospital (grant numbers TSGH-E-111229 and TSGH-E-112231).

### Institutional Review Board statement

The Ethical Review Board of Tri-Service General Hospital (Taiwan) approved the clinical studies (TSGHIRB No: B-110-57).

### Disclosures

The authors declare that there are no conflicts of interest related to this article.

## Supplementary Material

Supplementary data

## Figures and Tables

**Table 1:**
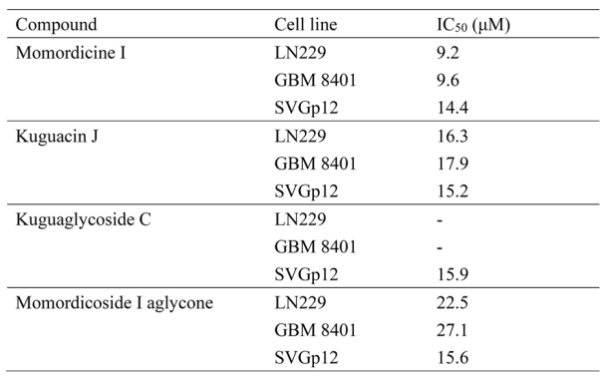
IC_50_ values of the indicated compounds in SVGp12 astrocytes and GBM8401 and LN229 glioma cells

**Figure 1:**
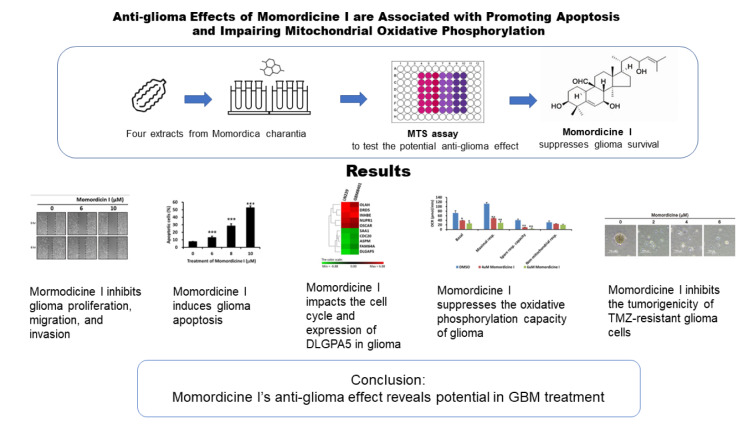
Graphical abstract
