# Supplementary material for: Momordicine I suppresses glioma growth by promoting apoptosis and impairing mitochondrial oxidative phosphorylation
Source: EXCLI J. 2023 Jun 6;22:482–98. doi: 10.17179/excli2023-6129 (PMC10391611; doi:10.17179/excli2023-6129)

EXCLI Journal 2023;22:482-498 – ISSN 1611-2156
Received: April 25, 2023, accepted: May 30, 2023, published: June 06, 2023

Supplementary data to:

Original article:

MOMORDICINE I SUPPRESSES GLIOMA GROWTH BY PROMOTING APOPTOSIS AND IMPAIRING MITOCHONDRIAL OXIDATIVE PHOSPHORYLATION

Ying Kao[1,2], Chung-Hsing Chou[3], Li-Chun Huang[4], Chia-Kuang Tsai[3*]

[1] Division of Neurosurgery, Department of Surgery, Taipei City Hospital Zhongxing Branch, Taipei 10341, Taiwan
[2] Taipei City University, Taipei 100234, Taiwan
[3] Department of Neurology, Tri-Service General Hospital, National Defense Medical Center, Taipei 11490, Taiwan
[4] Department of Biochemistry, National Defense Medical Center, Taipei 11490, Taiwan

* **Corresponding author:** Chia-Kuang Tsai, M.D. Ph.D., Department of Neurology, Tri-Service General Hospital, National Defense Medical Center, No. 325, Sec. 2, Cheng-gong Road, Taipei, Taiwan 11490. Tel: +886-2-87923311 ext 12860; Fax: +886-2-87927174, E-mail: jiakuang@mail.ndmctsgh.edu.tw

https://dx.doi.org/10.17179/excli2023-6129

This is an Open Access article distributed under the terms of the Creative Commons Attribution License (http://creativecommons.org/licenses/by/4.0/).

EXCLI Journal 2023;22:482-498 – ISSN 1611-2156
Received: April 25, 2023, accepted: May 30, 2023, published: June 06, 2023

Raw data of Figure 2a: Raw data of MTS assay showing the capacity of cell proliferation of LN229, GBM8401, and SVGp12 cells exposed to indicated dosage of Momordicin I for 48 hours.

Cell line	Experiment	Momordicine I (μM)						
		0	2	6	8	10	12	16
LN229	1	1.177	1.369	1.305	0.735	0.486	0.357	0.172
	2	1.405	1.506	1.327	0.884	0.538	0.341	0.200
	3	1.347	1.504	1.480	0.810	0.574	0.437	0.204
GBM8401	1	1.8998	1.9376	1.7607	1.2608	0.9011	0.6574	0.3513
	2	1.9233	1.8202	1.6997	1.1312	0.891	0.6123	0.3409
	3	1.8281	1.8202	1.7696	1.2141	0.8429	0.5983	0.28
SVGp12	1	0.485	0.468	0.490	0.488	0.434	0.323	0.166
	2	0.489	0.526	0.504	0.521	0.470	0.430	0.241
	3	0.480	0.548	0.545	0.484	0.447	0.322	0.188

Raw data of Figure 2b: Raw data of MTS assay showing the capacity of cell proliferation of LN229, GBM8401, and SVGp12 cells exposed to indicated dosage of Kuguacin J for 48 hours.

Cell line	Experiment	Kuguacin J (μM)						
		0	2	6	8	10	12	16
LN229	1	1.763	1.840	1.609	1.560	1.554	1.452	0.966
	2	1.770	1.781	1.656	1.461	1.487	1.253	0.897
	3	1.726	1.758	1.638	1.391	1.454	1.253	0.878
GBM8401	1	1.875	1.924	1.791	1.509	1.500	1.544	1.181
	2	1.869	1.850	1.729	1.484	1.523	1.545	1.116
	3	1.812	1.947	1.859	1.514	1.505	1.449	1.047
SVGp12	1	0.184	0.205	0.221	0.198	0.204	0.144	0.055
	2	0.158	0.145	0.220	0.188	0.190	0.143	0.065
	3	0.149	0.157	0.174	0.184	0.160	0.124	0.068

EXCLI Journal 2023;22:482-498 – ISSN 1611-2156
Received: April 25, 2023, accepted: May 30, 2023, published: June 06, 2023

Raw data of Figure 2c: Raw data of MTS assay showing the capacity of cell proliferation of LN229, GBM8401, and SVGp12 cells exposed to indicated dosage of Kuguaglycoside C for 48 hours.

Cell line	Experiment	Kuguaglycoside C (µM)						
		0	2	6	8	10	12	16
LN229	1	1.248	1.405	1.458	1.587	1.874	1.809	1.196
	2	1.294	1.433	1.558	1.673	1.763	1.662	1.292
	3	1.293	1.619	1.700	1.678	1.751	1.618	1.228
GBM8401	1	1.557	1.730	1.751	1.719	1.776	1.791	1.770
	2	1.599	1.659	1.735	1.794	1.901	1.852	1.610
	3	1.588	1.646	1.691	1.750	1.761	1.767	1.593
SVGp12	1	0.236	0.223	0.224	0.219	0.206	0.167	0.105
	2	0.211	0.211	0.250	0.213	0.195	0.178	0.117
	3	0.222	0.217	0.217	0.225	0.222	0.184	0.129

Raw data of Figure 2d: Raw data of MTS assay showing the capacity of cell proliferation of LN229, GBM8401, and SVGp12 cells exposed to indicated dosage of Momordicoside I aglycne for 48 hours.

Cell line	Experiment	Momordicoside I aglycne (µM)						
		0	2	6	8	10	12	16
LN229	1	1.669	1.786	1.685	1.678	1.676	1.641	1.202
	2	1.743	1.595	1.637	1.609	1.531	1.564	1.364
	3	1.702	1.572	1.529	1.531	1.528	1.593	1.377
GBM8401	1	1.753	1.677	1.683	1.810	1.773	1.846	1.611
	2	1.744	1.614	1.661	1.679	1.742	1.789	1.506
	3	1.816	1.704	1.730	1.885	1.730	1.753	1.547
SVGp12	1	0.160	0.177	0.200	0.184	0.163	0.125	0.051
	2	0.167	0.211	0.180	0.184	0.184	0.168	0.063
	3	0.149	0.146	0.190	0.174	0.184	0.146	0.076

EXCLI Journal 2023;22:482-498 – ISSN 1611-2156
Received: April 25, 2023, accepted: May 30, 2023, published: June 06, 2023

Raw data of Figure 3b: Raw data of colony formation assay showing the capacity of colony formation of LN229 and GBM8401 cells exposed to indicated dosage of Momordicine I for 14 days. The numbers of colonies were quantified by ImageJ software (NIH, Bethesda, MD) and colonies larger than 0.5 mm in diameter were counted.

**Cell line**	**Experiment**	**Momordicine I (μM)**			
		**0**	**4**	**6**	**8**
LN229	1	143	46	28	16
	2	133	38	31	18
	3	183	46	32	19
GBM8401	1	153	102	58	28
	2	216	97	44	24
	3	168	103	54	38

EXCLI Journal 2023;22:482-498 – ISSN 1611-2156
Received: April 25, 2023, accepted: May 30, 2023, published: June 06, 2023

Raw data of Figure 4a: Cell proliferation of LN229 and GBM8401 treated with Momordicine I for 48 hours. The data provided are the percentage of the positive BrdU incorporation cells at the specified concentration.

Cell line	Momordicine I (uM)	M2 (positive percentage)		
		1	2	3
LN229	0	31.6	32.8	30.8
	6	3.4	4.7	3.0
	8	1.2	2.3	1.7
	10	0.3	0.1	0.1
GBM8401	0	26.8	27.7	26.7
	6	5.5	4.1	4.7
	8	2.4	2.2	1.7
	10	0.7	0.6	1.0

Raw data of Figure 4b: Cellular apoptosis analysis of LN229 and GBM8401 cells was evaluated in flow cytometry with 7-AAD/PE Annexin V assay (BD MitoScreen), after Momordicine I therapy for 48 h.

Cell line	Momordicine I (uM)	Apoptotic cells (%) - UR+LR		
		1	2	3
LN229	0	4.14	5.22	5.64
	6	22.26	16.92	23.95
	8	40.14	38.03	44.98
	10	71.7	72.82	73.86
GBM8401	0	6.95	7.40	7.96
	6	13.09	10.99	14.79
	8	31.35	28.43	25.24
	10	54.07	49.74	54.04

EXCLI Journal 2023;22:482-498 – ISSN 1611-2156
Received: April 25, 2023, accepted: May 30, 2023, published: June 06, 2023

Raw data of Figure 4c: The Ki67 and Survivin expression in the LN229 and GBM8401 cells after 48 hours of treatment with indicated dose of momordicine I was quantified by densitometry evaluation using ImageJ software (National Institutes of Health, Bethesda, MD). These values were referenced to the values of ACTN of the same membrane as an internal control.

Cell line		Experiment	Momordicine I (μM)			
			0	6	8	10
**LN229**	Ki67	1	0.861	0.124	0.287	0.058
		2	0.279	0.056	0.075	0.101
		3	0.588	0.143	0.066	0.064
	Survivin	1	0.978	0.099	0.055	0.071
		2	0.505	0.047	0.057	0.059
		3	0.376	0.083	0.083	0.044
**GBM8401**	Ki67	1	1.042	0.635	0.144	0.066
		2	0.671	0.154	0.089	0.142
		3	0.699	0.222	0.121	0.125
	Survivin	1	1.090	0.155	0.051	0.023
		2	0.892	0.118	0.070	0.122
		3	0.816	0.120	0.142	0.122

EXCLI Journal 2023;22:482-498 – ISSN 1611-2156
Received: April 25, 2023, accepted: May 30, 2023, published: June 06, 2023

Raw images of the Western blot analyses for the Ki67, Suvivin and β-actin protein in the LN229 and GBM8401 cells (**Figure 4c**)

Experiment 1

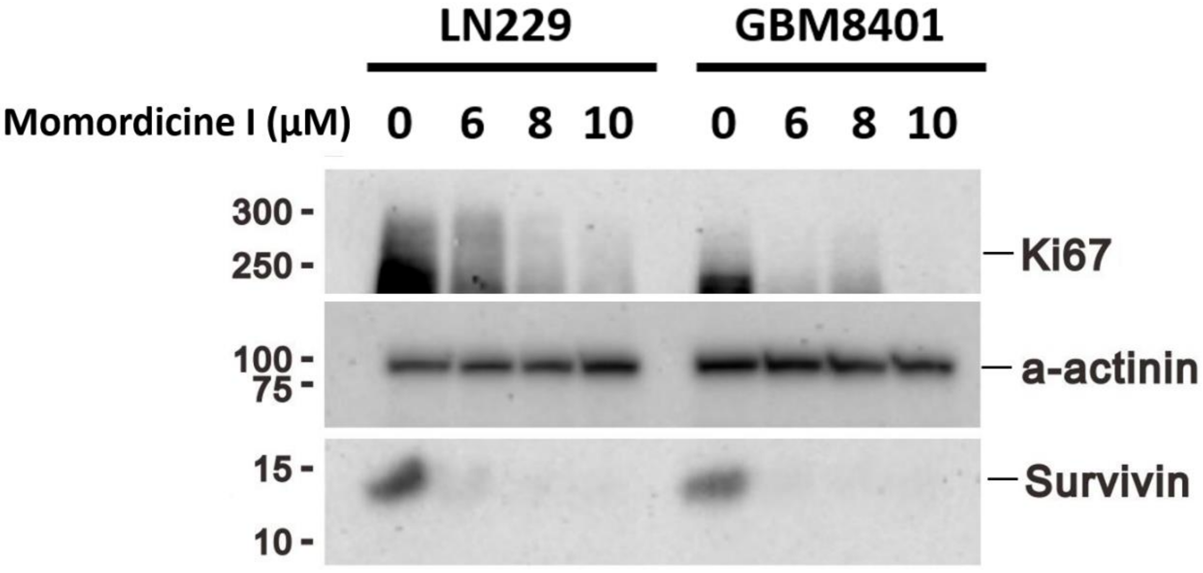

Experiment 2

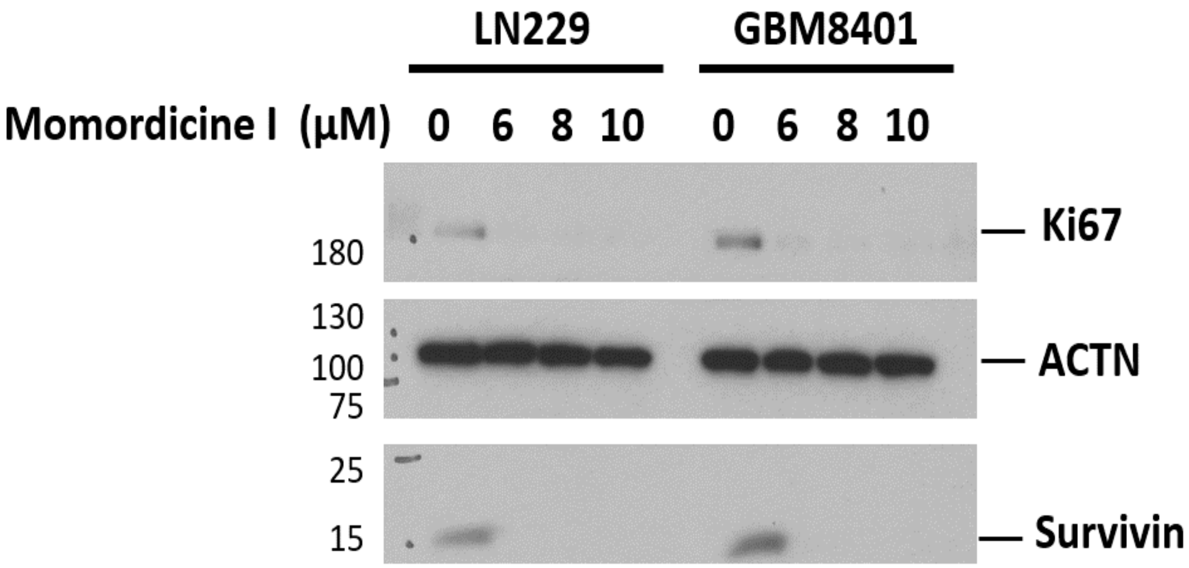

EXCLI Journal 2023;22:482-498 – ISSN 1611-2156
Received: April 25, 2023, accepted: May 30, 2023, published: June 06, 2023

Experiment 3

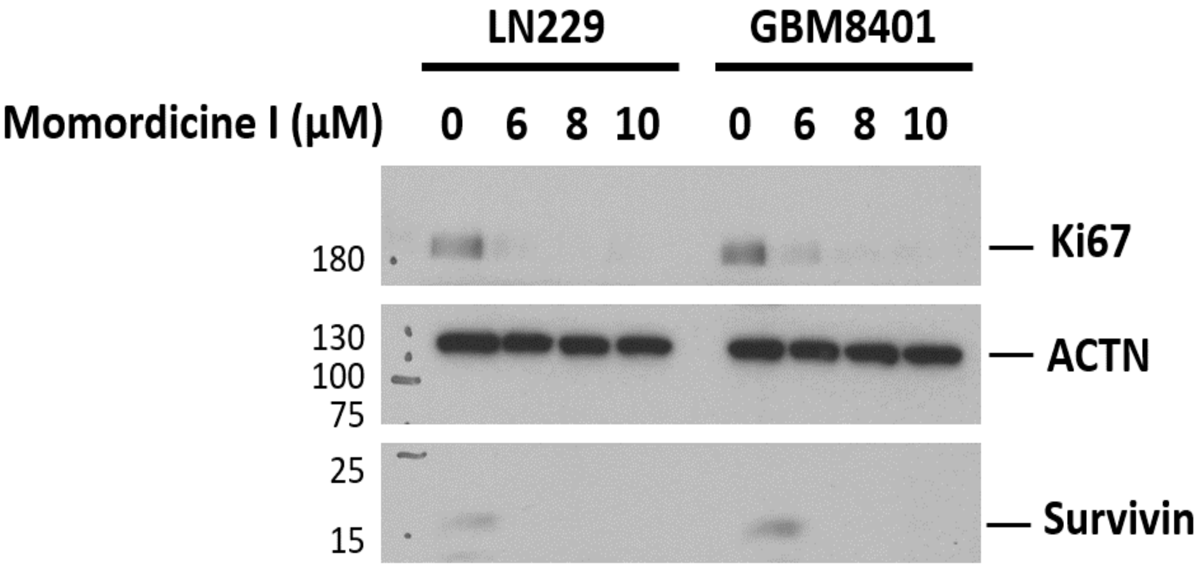

EXCLI Journal 2023;22:482-498 – ISSN 1611-2156
Received: April 25, 2023, accepted: May 30, 2023, published: June 06, 2023

Raw data of **Figure 5a:** Scratches were made in LN229 and GBM8401 glioma cell monolayers in 6-well plates, and the cells were cultured in the presence or absence of momordicine I for 16 h. Treatment with momordicine I (6 and 10 μM) significantly decreased the wound area relative to that in the control group in both LN229 and GBM8401 cells. All cells were pretreated with mitomycin C, a cell cycle inhibitor, to assess migration without the confounding impact of cell proliferation. The wound area was analyzed with ImageJ software, and data are expressed relative to hour 0.

Cell line	LN229			GBM8401		
Time	0 hr	16 hr		0 hr	16 hr	
Momordicine I (μM)	Area	Area	0 hr-16hr	Area	Area	0 hr-16hr
0	1017658	575085	442573	1217286	568161	649125
0	1263989	771021	492968	1261961	652247	609714
0	1286871	826482	460389	1162942	556489	606453
6	1408973	1039987	368986	1292349	698057	594292
6	1410915	979613	431302	1314590	719883	594707
6	1406717	986477	420240	1316358	814245	502113
10	1588087	1428634	159453	1385464	968996	416468
10	1864943	1570293	294650	1495779	1035533	460246
10	1586774	1374927	211847	1421927	1005231	416696

Raw data of Figure 5b: To assess the cell invasive ability, we performed the transwell invasion assays. LN229 and GBM8401 cells were seeded in serum-free DMEM with or without momordicine I into the top chambers of transwell (BD Biosciences) whereas lower chamber consisted of DMEM with 2 % FBS serving as a chemoattractant for the cells. Following incubating the chambers for 16 h, glioma cells in the upper surface of chambers had migrated to the lower side of the membrane. These cells invaded through matrigel at the lower surface were fixed in 4 % formaldehyde and stained with 0.1 % crystal violet (Merck Millipore). Quantification of invasive cells was done by counting in three arbitrarily selected fields in each membrane.

Cell line	Momordicine I (μM)	Experiment		
		1	2	3
LN229	0 mM	0.3642	0.6070	0.4268
	6 mM	0.3280	0.3201	0.3455
	10 mM	0.2461	0.2182	0.1831
GBM8401	0 mM	0.4168	0.4985	0.4841
	6 mM	0.4144	0.5310	0.2170
	10 mM	0.1288	0.3261	0.2459

Raw data of Figure 5c: The N-cadherin and Twist protein levels in LN229 and GBM8401 cells treated with 6, 8, and 10 μM momordicine I for 48 h were quantified by densitometry evaluation using ImageJ software. These values were referenced to the internal control β-actin values.

Cell line	N-cadherin Experiment	Momordicine I (μM)			
		0	6	8	10
LN229	1	0.3736	0.1814	0.1493	0.1835
	2	0.65844	0.60777	0.6604	0.67252
	3	0.47398	0.5079	0.46026	0.49921
GBM8401	1	0.63159	0.46521	0.41779	0.29394
	2	1.02468	0.88332	0.86751	0.80999
	3	0.92764	0.81879	0.72458	0.70687

Cell line	Twist Experiment	Momordicine I (μM)			
		0	6	8	10
LN229	1	0.6497	0.4802	0.3388	0.3039
	2	1.10989	1.54987	0.90777	0.92487
	3	1.62179	1.36331	1.23816	1.51244
GBM8401	1	0.6175	0.30802	0.33768	0.2878
	2	0.9954	0.7409	0.57044	0.64052
	3	1.41815	1.13542	1.13408	1.15115

EXCLI Journal 2023;22:482-498 – ISSN 1611-2156
Received: April 25, 2023, accepted: May 30, 2023, published: June 06, 2023

Raw images of the Western blot analyses for the N-cadherin, Twist and β-actin protein in the LN229 and GBM8401 cells (**Figure 5c**)

Experiment 1

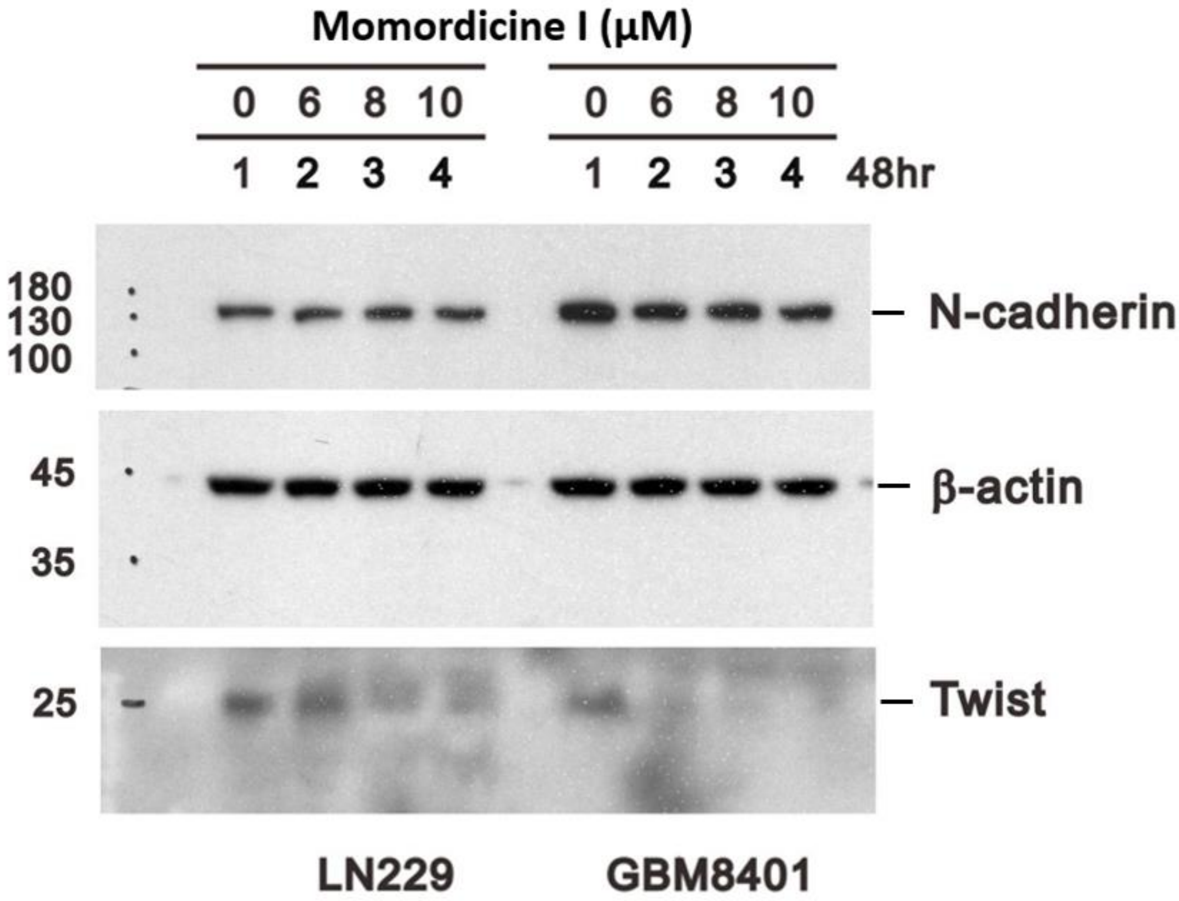

EXCLI Journal 2023;22:482-498 – ISSN 1611-2156
Received: April 25, 2023, accepted: May 30, 2023, published: June 06, 2023

Experiment 2

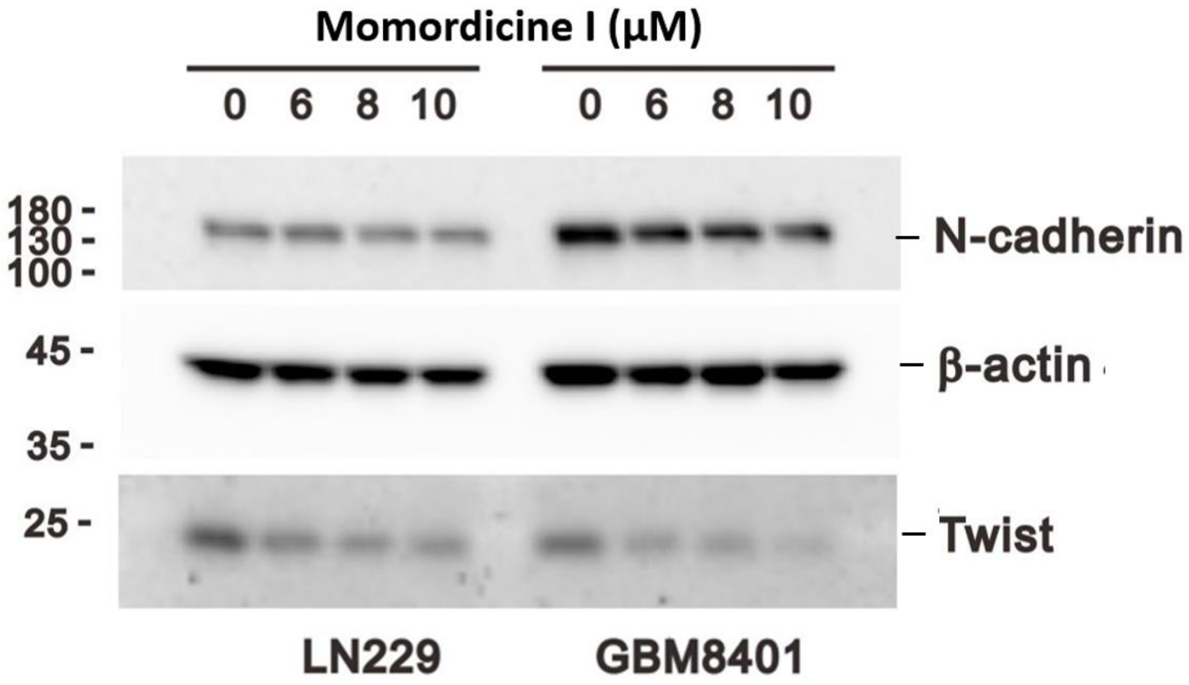

Experiment 3

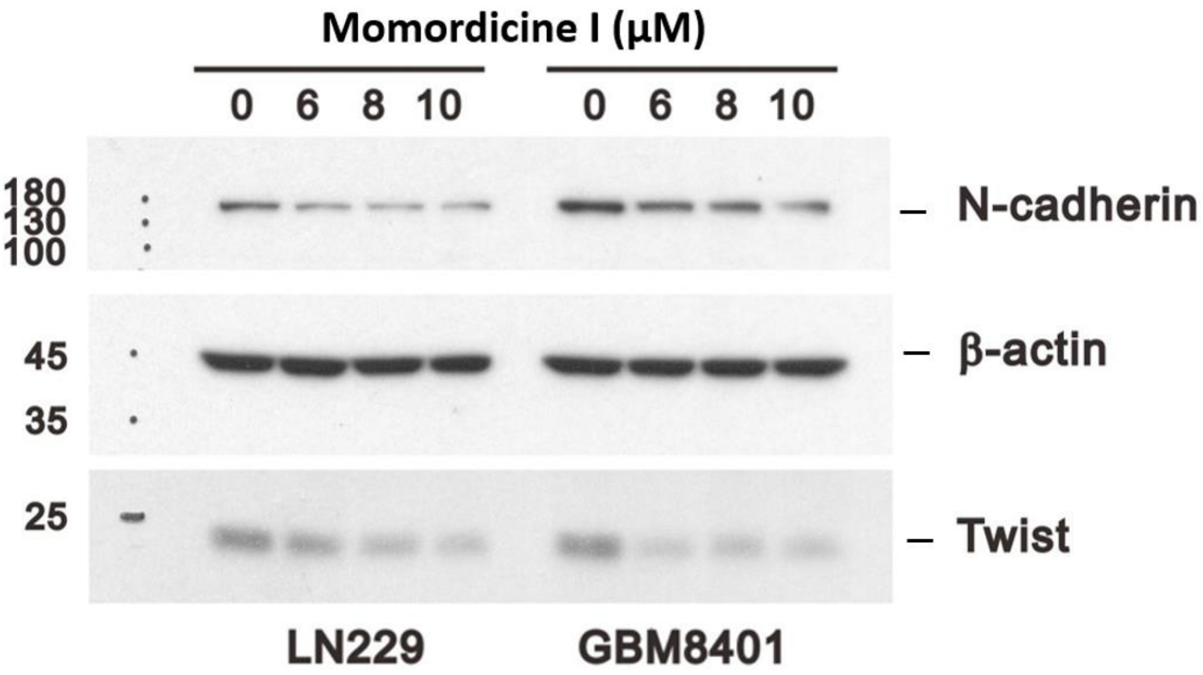

EXCLI Journal 2023;22:482-498 – ISSN 1611-2156
Received: April 25, 2023, accepted: May 30, 2023, published: June 06, 2023

Raw data of Figure 7: To measure cellular oxidative respiration, a Seahorse XF bioenergetic system was utilized in combination with a Seahorse Cell Mito Stress Test Kit (Seahorse Bioscience, North Billerica, MA, USA). LN229 and GBM8401 glioma cells were seeded into an XFp microplate in DMEM supplemented with 2 % FBS and treated with momordicine for 24 hours over the following two days.

Cell line		LN229		GBM8401	
Experiment	Substance	OCR (pmol/min)			
		1st	2nd	1st	2nd
Measurement 1	DMSO	100.948	128.294	224.658	242.740
	Momordicine I 4 µM	72.811	74.915	177.773	193.877
	Momordicine I 6 µM	58.958	46.974	127.803	105.178
Measurement 2	DMSO	94.404	118.047	208.889	229.283
	Momordicine I 4 µM	66.083	68.664	167.006	177.792
	Momordicine I 6 µM	54.260	42.990	119.340	98.850
Measurement 3	DMSO	92.269	115.597	205.226	225.729
	Momordicine I 4 µM	62.912	66.155	163.446	172.430
	Momordicine I 6 µM	51.642	41.141	116.036	97.508
Measurement 4	DMSO	91.534	114.902	202.269	223.518
	Momordicine I 4 µM	61.897	64.599	160.996	168.949
	Momordicine I 6 µM	50.860	40.236	113.996	95.619
Measurement 5	DMSO	72.369	98.272	110.681	123.073
	Momordicine I 4 µM	50.580	55.355	89.361	133.710
	Momordicine I 6 µM	45.338	37.447	72.149	59.967
Measurement 6	DMSO	51.325	78.011	104.743	114.903
	Momordicine I 4 µM	41.683	43.622	90.083	101.389
	Momordicine I 6 µM	37.988	33.400	72.905	60.617
Measurement 7	DMSO	43.741	65.617	103.238	112.244
	Momordicine I 4 µM	38.081	40.297	89.836	98.316
	Momordicine I 6 µM	33.465	30.131	73.290	61.161
Measurement 8	DMSO	135.832	152.014	291.856	305.330
	Momordicine I 4 µM	71.822	74.428	220.639	227.776
	Momordicine I 6 µM	56.441	41.046	151.206	127.675
Measurement 9	DMSO	95.118	108.203	242.961	258.991
	Momordicine I 4 µM	55.036	57.606	183.335	183.968
	Momordicine I 6 µM	52.375	38.108	119.579	100.116
Measurement 10	DMSO	76.322	88.386	225.582	243.746
	Momordicine I 4 µM	47.706	49.985	167.600	167.033
	Momordicine I 6 µM	48.276	35.326	109.181	91.042
Measurement 11	DMSO	28.477	37.408	88.523	93.574
	Momordicine I 4 µM	25.274	26.978	73.503	80.006
	Momordicine I 6 µM	22.769	18.842	62.797	52.332
Measurement 12	DMSO	28.191	37.939	85.704	90.813
	Momordicine I 4 µM	24.610	27.057	73.087	78.777
	Momordicine I 6 µM	22.317	18.762	62.094	52.144
Measurement 13	DMSO	27.232	35.675	82.972	89.918
	Momordicine I 4 µM	23.354	24.715	72.411	77.726
	Momordicine I 6 µM	21.867	17.543	60.657	50.957

EXCLI Journal 2023;22:482-498 – ISSN 1611-2156
Received: April 25, 2023, accepted: May 30, 2023, published: June 06, 2023

Raw data of Figure 8b: GBM1 cells, $7.5*10^4$ cells/well, were cultured in DMEM with 2 % FBS in an Ultra-Low 6-well plate and treated with Momordicine I for 14 days. Only tumor sphere greater than 50 µm were counted.

Momordicine I (µM)	Number of spheres/well		
0	10	14	8
2	0	0	0
4	1	0	0
6	0	0	0

Raw data of Figure 8b: GBM2 cells, $5*10^4$ cells/well, were cultured in DMEM with 2% FBS in an Ultra-Low 6-well plate and treated with Momordicine I for 14 days. Only tumor sphere greater than 50um were counted.

Momordicine I (µM)	Number of spheres/well		
0	23	14	17
4	8	12	19
6	9	10	16
8	1	12	3

Raw data of Figure 8b: GBM3 cells, $5*10^4$ cells/well, were cultured in DMEM with 2 % FBS in an Ultra-Low 6-well plate and treated with Momordicine I for 14 days. Only tumor sphere greater than 50 µm were counted.

Momordicine I (µM)	Number of spheres/well		
0	27	27	28
4	25	27	28
6	17	19	18
8	10	10	11

Received: April 25, 2023, accepted: May 30, 2023, published: June 06, 2023

Raw data of Figure 9c: Cell-cycle analysis by flow cytometry of LN229 cells treated with various dosage of momordicine I for 48 hours. The data given are values obtained from the PI staining.

LN229	Momordicine I (µM)							
**Cell cycle phase (%)**	**0**	**0**	**6**	**6**	**8**	**8**	**10**	**10**
subG1	0.52	0.38	0.25	0.45	0.36	0.63	1.1	0.7
G1	84.83	81.97	78.64	78.83	87.59	86.75	92.97	93.70
S	5.28	5.98	8.45	5.83	3.38	3.84	1.06	0.67
G2/M	9.45	11.76	12.75	13.17	8.72	8.86	4.91	4.93

Raw data of Figure 9c: Cell-cycle analysis by flow cytometry of GBM8401 cells treated with various dosage of momordicine I for 48 hours. The data given are values obtained from the PI staining.

GBM8401	Momordicine I (uM)							
**Cell cycle phase (%)**	**0**	**0**	**6**	**6**	**8**	**8**	**10**	**10**
subG1	0.48	0.41	0.44	0.39	1.1	0.5	0.46	0.4
G1	74.55	76.68	74.21	73.67	80.08	82.26	87.03	88.52
S	8.55	8.88	7.22	7.96	3.97	3.22	1.48	1.20
G2/M	16.53	14.09	18.2	18.14	11.98	14.06	8.55	9.90

Raw data of Figure 9f: The DLGAP5 protein levels in LN229 and GBM8401 cells treated with 6, 8, and 10 µM momordicine I for 48 h was quantified by densitometry evaluation using ImageJ software. These values were referenced to the values of GAPDH of the same membrane as an internal control.

Cell line	Experiment	Momordicine I (µM)			
		**0**	**6**	**8**	**10**
LN229	1	0.397	0.233	0.259	0.241
	2	0.296	0.297	0.255	0.234
	3	0.089	0.042	0.020	0.013
GBM8401	1	0.459	0.323	0.285	0.315
	2	0.103	0.055	0.016	0.015
	3	0.078	0.032	0.021	0.027

EXCLI Journal 2023;22:482-498 – ISSN 1611-2156
Received: April 25, 2023, accepted: May 30, 2023, published: June 06, 2023

Raw images of the Western blot analyses for the DLGAP5 and GAPDH protein in the LN229 and GBM8401 cells (**Figure 9c**)

Experiment 1

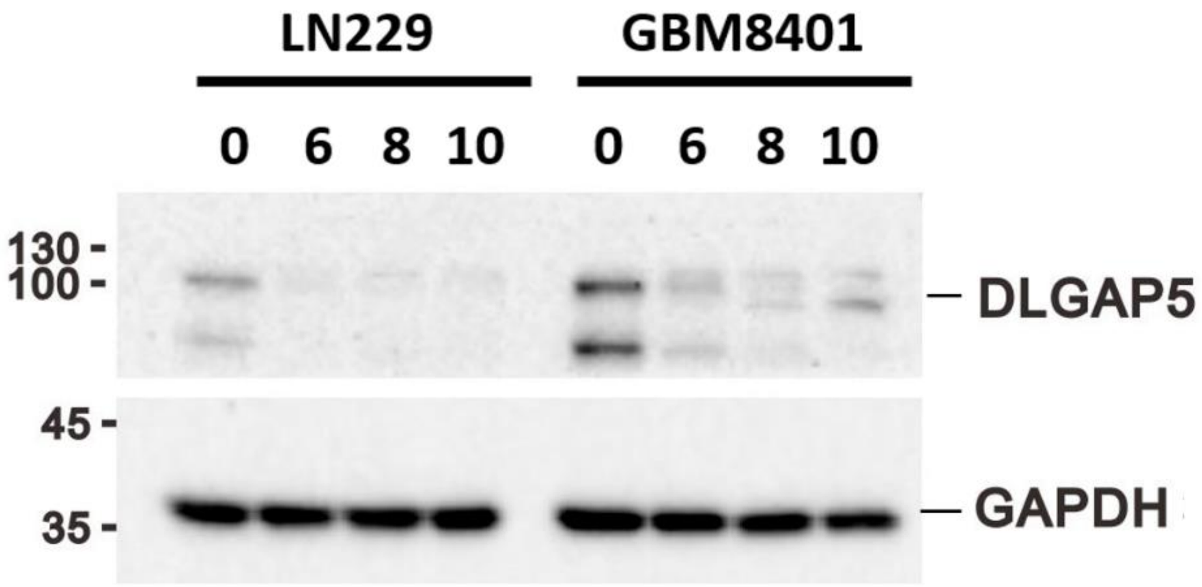

Experiment 2

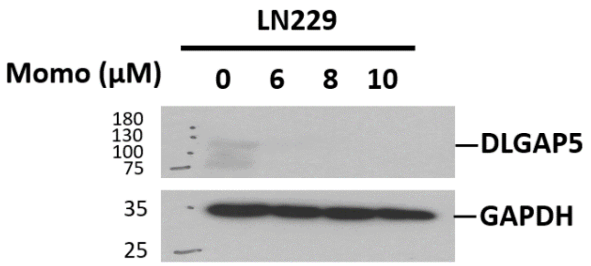

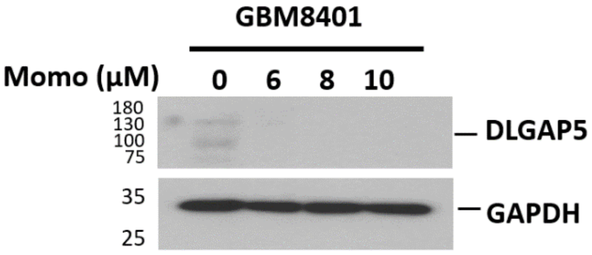

Experiment 3

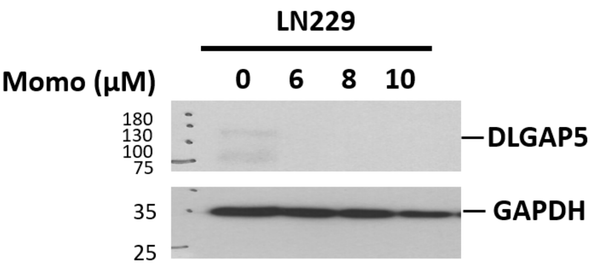

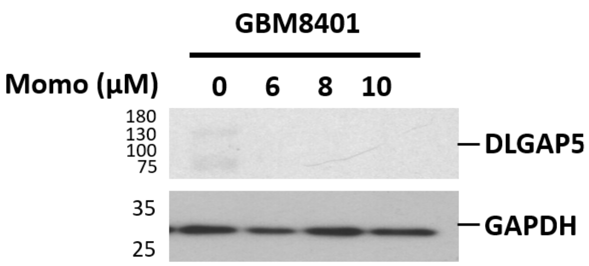

Supplement: Supplementary data [file EXCLI-22-482-s-001.pdf]
